# Square dance participation and subjective well-being among urban older adults: mediating roles of social support and optimism

**DOI:** 10.3389/fpsyg.2026.1893996

**Published:** 2026-08-06

**Authors:** Longan Cao, Zhirong Zheng, Jun Lang, Zhengban Ran, Lei Wang

**Affiliations:** 1 School of Physical Education, Southwest University, Chongqing, China; 2 Teaching and Research Association, Chongqing Vocational College of Applied Technology, Chongqing, China; 3 Teaching and Research Association, Guang’an Institute of Technology, Guang’an, Sichuan, China

**Keywords:** chain mediation, older adults, optimism, social support, square dance participation, subjective well-being

## Abstract

**Background:**

Promoting a happier society is an important goal of social development, and subjective well-being is a key indicator of quality of life. With population aging in China, older adults may be more vulnerable to negative emotional states. Group-based physical activities, such as square dance, have become popular among older adults due to their accessibility and potential psychological benefits.

**Methods:**

Using a convenience sampling approach, this study recruited urban older adults aged 60–69 years who regularly participated in square dance exercise. Notably, the final sample was predominantly female (94.55%), which should be considered when interpreting the findings. Data were collected using validated scales, including the PARS-3, Social Support Scale, LOT-R, and the Chinese Urban Residents’ Subjective Well-being Scale (short version). Analyses were conducted using SPSS 27.0 and AMOS 26.0, and mediation effects were tested using bootstrap analysis.

**Results:**

Square dance participation, social support, optimism, and subjective well-being were significantly positively associated (*p* < 0.01). Square dance participation was associated with subjective well-being (total effect β = 0.507), with a direct effect of 0.205. Social support partially mediated this relationship (indirect effect = 0.233), as did optimism (indirect effect = 0.034). A sequential mediation effect through social support and optimism was also observed (indirect effect = 0.035).

**Conclusion:**

Square dance participation is positively associated with subjective well-being among older adults, with this relationship partially explained by social support and optimism.

## 1 Introduction

Health is widely recognized as a fundamental human right and a key foundation of social development. In recent years, population aging has become a global challenge, particularly in rapidly urbanizing countries such as China. According to recent demographic data, older adults account for a substantial proportion of the urban population, with more than half of older individuals residing in urban areas (Wang et al., 2025). Aging is often accompanied by declines in physiological function and an increased risk of chronic diseases, which may be associated with negative emotional states and reduced well-being (Matteson et al., 2022). Consequently, promoting subjective well-being among older adults has become an important public health concern in aging societies (Mao and Han, 2018).

Research on subjective well-being dates back to the 1950s and has since become a central topic in positive psychology. It is widely regarded as an important indicator of individuals’ social functioning, psychological adaptation, and overall quality of life. With the rise of positive psychology in the 1990s, subjective well-being received increasing attention as a core construct in the field (Mio, 2003). Diener provided one of the most influential definitions, describing subjective well-being as individuals’ cognitive and affective evaluations of their lives (Diener, 1984). This construct includes both life satisfaction (a cognitive component) and emotional experiences (an affective component) (Blasco-Belled et al., 2018). In line with this framework, the present study defines subjective well-being as individuals’ overall evaluation of their life satisfaction and perceived quality of life within a given context.

According to the United Nations definition proposed at the World Assembly on Aging (1982), individuals aged 60 years and above are generally classified as older adults, although the threshold may vary between 60 and 65 years across different contexts. In China, 60 years is commonly adopted as the starting point for older adulthood, considering the national demographic and social context. In the present study, individuals aged 60–69 years were selected as the target population. This group, often referred to as the “young-old,” typically represents the early stage of older adulthood and is frequently characterized by the transition into retirement (Richardson, 2003). Such transitions are often accompanied by changes in social roles and emotional states, which may render this population particularly vulnerable to fluctuations in psychological well-being (Nakray, 2022; Liu, 2025). Therefore, focusing on this age group provides a meaningful context for examining factors associated with subjective well-being.

Square dance is a form of group-based physical activity performed in public spaces with music accompaniment and has become increasingly popular among older adults in China due to its accessibility and low physical demands (Huang, 2022; Hu, 2022). Previous studies indicate that this light-intensity aerobic activity is suitable for middle-aged and older populations, as it can be performed in community settings with minimal equipment or space requirements (Jiang and Ra, 2017; Tsai et al., 2021; Kobayashi and Negoro, 2024). From a psychosocial perspective, participation in group-based physical activities has been associated with physical and psychological outcomes in older adults. Prior research has reported associations between square dance participation and indicators such as physical fitness, weight status (Todorovich, 2012), and muscle function (Schulze and Möhwald, 2025). In addition, such activities have been linked to reduced depressive symptoms and improved quality of life among older adults (Irani et al., 2020; Ou et al., 2022). Group-based activities may also facilitate social interaction and engagement in daily life, which may be related to higher levels of subjective well-being. However, the mechanisms underlying the association between square dance participation and subjective well-being remain insufficiently explored. In particular, it is unclear whether this relationship is direct or operates through psychosocial factors such as social support and optimism. Therefore, further research is needed to examine potential mediating pathways linking group-based physical activity and subjective well-being in older adults.

Social support is a well-established construct in psychological research. Cobb (1976) described social support as information that leads individuals to believe that they are cared for, loved, valued, and part of a network of mutual obligation. Bierhoff (1994) further emphasized social support as the perception of being loved, valued, and supported within one’s social network (Bierhoff, 1994). A growing body of evidence suggests that social support plays a crucial role in promoting psychological well-being and reducing negative emotional states (Lam, 2018). In older adults, higher levels of perceived social support have been associated with greater life satisfaction and lower levels of depressive symptoms (Ioannou et al., 2019). Moreover, social support and physical activity appear to be mutually reinforcing. For example, Lindsay Smith found that social support from family and friends is a key factor influencing older adults’ participation in physical activity, particularly during leisure time (Lindsay Smith et al., 2017). As a form of group-based physical activity, square dance provides a socially engaging environment that facilitates interpersonal interaction and social network expansion (Banio-Krajnik, 2023). Participation in such activities may enhance individuals’ sense of belonging and perceived support from others, including family members, friends, and peers (Langford et al., 2024). In turn, increased social support may contribute to improved subjective well-being. Therefore, social support is likely to serve as an important mediating factor in the relationship between square dance participation and subjective well-being among older adults (Mao, 2021).

Optimism is widely recognized as an important psychological resource that contributes to both physical and mental health. A growing body of research suggests that engagement in physical activity is positively associated with the development of optimistic dispositions (Song and Lee, 2021). For instance, individuals who engage less frequently in physical activity tend to report lower scores on measures of dispositional optimism (Stryapukhina, 2026). In older adults, optimism has been shown to be a significant predictor of subjective well-being and overall mental health. Moreover, optimism has been found to interact with other psychosocial factors, such as social support and self-efficacy, in enhancing psychological resilience and well-being among older populations. As a form of accessible group-based physical activity, square dance may contribute to the development of positive emotional experiences and foster optimistic outlooks (Cho et al., 2023). In turn, increased optimism may enhance individuals’ subjective well-being by promoting adaptive coping and positive life evaluations (Frydenberg, 2018). However, despite the established associations between physical activity, optimism, and well-being, limited research has examined whether optimism serves as an independent mediating factor in this relationship. Therefore, the present study aims to explore the potential mediating role of optimism in the association between square dance participation and subjective well-being among older adults.

A growing body of research has examined the relationship between social support and optimism, suggesting that these two psychosocial factors are closely interconnected. Social support, as an external resource, has been shown to play a significant role in shaping individuals’ psychological dispositions (Udry, 2001), including optimism. Empirical studies have demonstrated that higher levels of perceived social support are associated with greater optimism and more positive emotional experiences (Dawson and Pooley, 2013). In addition, the interaction between social support and optimism has been found to be particularly important for mental health outcomes. For instance, social support may enhance individuals’ psychological resources, enabling them to cope more effectively with stress and adversity (Carlson, 2016), thereby fostering a more optimistic outlook. Conversely, individuals with higher levels of optimism are more likely to perceive and utilize available social support, forming a mutually reinforcing relationship between these two factors (Vollmann et al., 2005). Importantly, both social support and optimism have been identified as correlates of psychological well-being and protective factors against negative emotional states, including depressive symptoms. These findings suggest that social support may be indirectly associated with subjective well-being through optimism. Therefore, social support and optimism are proposed to form a potential sequential mediation pathway linking group-based physical activity and subjective well-being among older adults.

**FIGURE 1:**
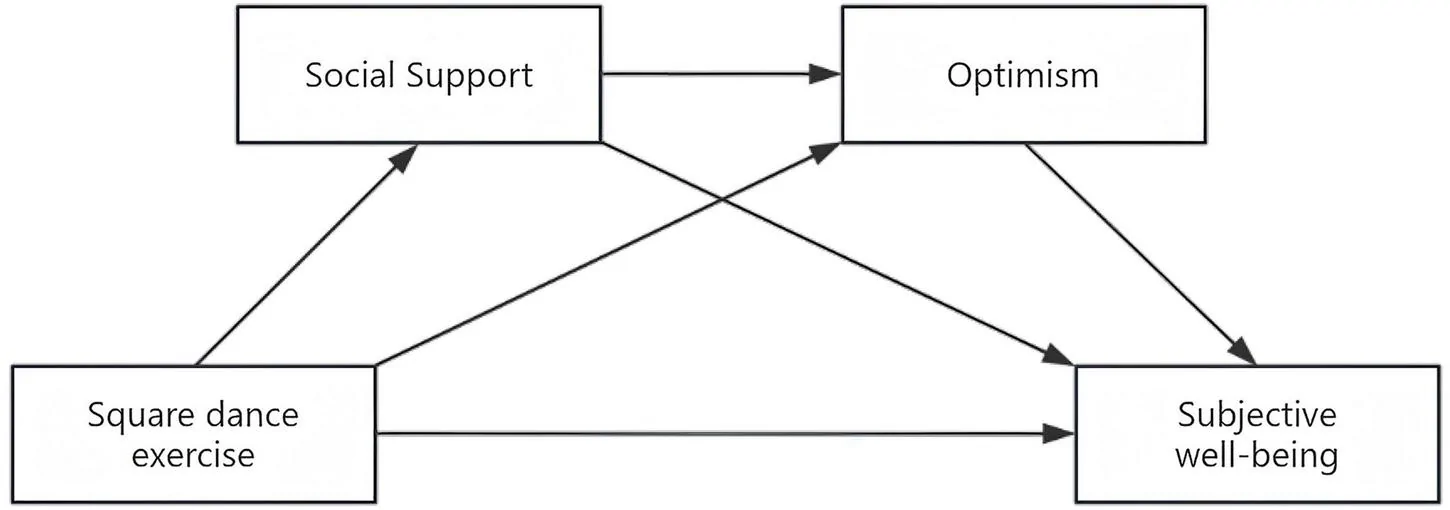
Assumed model diagram.

According to Diener’s theory of subjective well-being, individuals’ well-being is associated with multiple factors, including social relationships and personal characteristics. Previous research has reported that physical activity is associated with subjective well-being and may be related to psychosocial factors such as social support and optimism. From the perspective of social support theory, social support has been positively associated with optimism, and both constructs have been linked to mental health outcomes. In addition, theoretical frameworks such as Maslow’s hierarchy of needs suggest that fulfilling basic psychological needs through behavioral engagement (e.g., physical activity) may be related to higher levels of well-being (Roy, 2021; Strelicz and Berényi, 2025). Based on these theoretical perspectives and previous empirical findings, it is reasonable to propose that square dance participation may be associated with subjective well-being both directly and indirectly through psychosocial factors. In particular, social support and optimism are proposed as potential mediating variables, and social support may also be associated with optimism. Therefore, the present study aimed to examine the association between square dance participation and subjective well-being among urban older adults, as well as the potential mediating roles of social support and optimism, including their sequential mediation. The proposed hypotheses are presented in Figure 1.

H1: Square dance participation is positively associated with subjective well-being among urban older adults.

H2: Social support mediates the relationship between square dance participation and subjective well-being.

H3: Optimism mediates the relationship between square dance participation and subjective well-being.

H4: Social support is positively associated with optimism.

H5: Social support and optimism sequentially mediate the relationship between square dance participation and subjective well-being.

## 2 Materials and methods

### 2.1 Participants and procedure

Based on the research objectives, this study employed a convenience sampling approach to recruit urban older adults aged 60–69 years in Chongqing, China. Participants were recruited from community square dance activity sites and related social groups, and the sample therefore consisted exclusively of individuals who were actively engaged in square dance participation. No formal sampling frame or randomization procedure was applied in the recruitment process, and no non-participant or lapsed-participant comparison group was included. A total of 3,000 questionnaires were distributed, of which 2,832 were returned. After data screening, including data cleaning, transformation, and handling of missing values, 99 invalid responses were excluded, resulting in 2,733 valid questionnaires. The final response rate was 96.5%. The study involving human participants was reviewed and approved by the Ethics Committee at South West University (Approval Number 20250324J9). Written informed consent was obtained from all participants prior to data collection. Upon completion of the survey, a small token of appreciation (a commemorative gift) was provided to participants in recognition of their contribution. Detailed demographic characteristics are presented in Table 1.

**TABLE 1:** Demographic characteristics.

Variable	Category	*N*	Percentage (%)
Gender	Male	149	5.45
	Female	2584	94.55
Marital status	Married	2320	84.9
	Unmarried	74	2.7
	Widowed	126	4.6
	Divorced	213	7.8
Monthly income (RMB)	< 5000	533	19.5
	5000–10000	1708	62.5
	> 10000	492	18.0
Education level	Primary school or below	232	8.5
	Junior high school	842	30.8
	High school or vocational	1238	45.3
	College or above	421	15.4

### 2.2 Materials

#### 2.2.1 Square dance participation

Square dance participation was operationalized using the Physical Activity Rating Scale-3 (PARS-3) (Liang, 1994), which evaluates physical activity based on exercise intensity, duration, and frequency. The scale primarily captures the volume of physical activity rather than its social or interactional components. Therefore, it does not directly measure the social engagement or group interaction aspects that are conceptually associated with square dance participation. Because all participants in the present study were regular square dance practitioners, the PARS-3 score was used to reflect the level of participation in square dance rather than general physical activity. The total score is calculated as intensity × duration × frequency, ranging from 0 to 100, with higher scores indicating higher levels of physical activity. According to established criteria, scores ≤ 19 indicate low levels of physical activity, scores between 20 and 42 indicate moderate levels, and scores ≥ 43 indicate high levels of physical activity. In the present study, PARS-3 was used to assess the level of square dance participation. The internal consistency of the scale was acceptable (Cronbach’ s α = 0.825).

#### 2.2.2 Subjective well-being

Subjective well-being was assessed using the Chinese Urban Residents’ Subjective Well-being Scale developed by Xing (2003), which has been widely used in Chinese populations. The scale consists of 20 items and adopts a 6-point Likert response format. Among them, items 4, 5, 6, 9, 10, 11, 13, 15, 17, 18, and 20 are reverse-coded. The total score ranges from 20 to 120, with higher scores indicating higher levels of subjective well-being. According to established criteria, scores above 90 indicate high well-being, scores between 50 and 90 indicate moderate well-being, and scores below 50 indicate low well-being. The internal consistency of the scale in the present study was excellent (Cronbach’s α = 0.968).

#### 2.2.3 Optimism

Optimism was assessed using the Life Orientation Test-Revised (LOT-R) developed by Scheier, a widely used instrument for measuring dispositional optimism (Scheier et al., 1994). The scale consists of 10 items, including 6 substantive items and 4 filler items, with the latter not included in the scoring. Responses are rated on a 5-point Likert scale. Items 2, 4, and 5 are reverse-coded. Higher total scores indicate higher levels of optimism. The internal consistency of the scale in the present study was good (Cronbach’s α = 0.899).

#### 2.2.4 Social support

Social support was assessed using the Social Support Scale developed by Chen and revised by Duan (2019), which has been validated in Chinese populations. The scale consists of 17 items across four dimensions: family support (items 1–4), friend support (items 5–9), instrumental support (items 10–14), and informational support (items 15–17). All items are rated on a Likert scale, with higher total scores indicating higher levels of perceived social support. The internal consistency of the scale in the present study was excellent (Cronbach’s α = 0.917).

### 2.3 Data analysis

Statistical analysis was conducted using IBM SPSS 27.0 and AMOS 26.0. First, tests for normality and common method bias were performed to ensure data quality. Second, descriptive statistics, including means and standard deviations, were calculated for all variables. Third, independent samples *t*-tests and one-way analysis of variance (ANOVA) were conducted to examine differences in subjective well-being across demographic characteristics. Pearson’s correlation analysis was then used to examine bivariate relationships among the study variables. Finally, structural equation modeling (SEM) was conducted in AMOS 26.0 to test the hypothesized relationships among square dance participation, social support, optimism, and subjective well-being. A bootstrap procedure with 5,000 resamples was used to estimate indirect effects and their 95% confidence intervals (CIs). An effect was considered statistically significant if the confidence interval did not include zero (Rockwood and Hayes, 2020).

## 3 Results

### 3.1 Common method bias tests

Given that all variables were collected using self-reported questionnaires at a single time point, common method bias may exist. To mitigate this issue, both procedural and statistical remedies were applied. Procedurally, anonymity and confidentiality were ensured to reduce social desirability bias. Statistically, Harman’s single-factor test was conducted as an initial diagnostic technique. The results showed that seven factors had eigenvalues greater than 1, and the first factor accounted for 37.94% of the total variance, below the commonly referenced threshold of 40%. Although this suggests that common method bias is unlikely to be severe, Harman’s test alone has limitations and should be interpreted with caution.

### 3.2 Correlation analysis

Descriptive statistics and Pearson’s correlation coefficients for all study variables are presented in Table 2. The mean score of square dance participation was 42.69 ± 35.31, indicating a moderate level of physical activity among the participants. The relatively large standard deviation, which is close to the mean, indicates substantial inter-individual variability in physical activity participation among participants, suggesting a highly dispersed distribution. The mean levels of social support, optimism, and subjective well-being were all at moderate to relatively high levels. Correlation analysis revealed that square dance participation was positively correlated with social support, optimism, and subjective well-being (all *p* < 0.01). In addition, all dimensions of social support (family support, friend support, instrumental support, and informational support) were positively correlated with both optimism and subjective well-being (all *p* < 0.01). Furthermore, optimism was significantly positively correlated with subjective well-being (*p* < 0.01).

**TABLE 2:** Descriptive statistics and correlations among study variables (*N* = 2,733).

Variable	M ± SD	1	2	3	4	5	6	7
1. Square dance participation	42.69 ± 35.31	1	1	1	1	1	1	1
2. Family support	14.42 ± 4.63	0.36**						
3. Friend support	18.26 ± 5.47	0.39**	0.52**					
4. Instrumental support	18.24 ± 5.13	0.34**	0.48**	0.49**				
5. Informational support	10.69 ± 3.72	0.36**	0.45**	0.47**	0.44**			
6. Optimism	22.24 ± 6.16	0.44**	0.36**	0.38**	0.33**	0.37**		
7. subjective well-being	74.67 ± 24.41	0.46**	0.46**	0.43**	0.38**	0.36**	0.43**	

**p < 0.01.

### 3.3 Regression analysis

The results of the regression analyses are presented in Table 3. After controlling for demographic variables (gender, marital status, income, and education level), square dance participation was found to be a significant positive predictor of subjective well-being (β = 0.110, *p* < 0.001).

**TABLE 3:** Results of regression analyses.

Outcome variable	Predictor	R^2^	Adjusted R^2^	F	B	β	t
Subjective well-being	Gender	0.324	0.323	98.813***	−0.076	−0.075	−1.844
	Marital status				0.014	0.016	0.423
	Income				−0.079	−0.077	−2.049*
	Education level				−0.086	−0.089	−1.247
	Square dance participation				0.114	0.110	6.013***
Social support	Gender	0.366	0.361	65.440***	0.000	0.000	0.009
	Marital status				−0.063	−0.060	−1.763
	Income				0.057	0.055	1.298
	Education level				0.041	0.043	0.586
	Square dance participation				0.554	0.555	8.500***
Optimism	Gender	0.279	0.270	31.122***	−0.075	−0.073	−1.970*
	Marital status				0.026	0.026	1.324
	Income				−0.008	−0.008	−0.167
	Education level				−0.036	−0.035	−0.841
	Square dance participation				0.035	0.034	7.098***
	Social support				0.562	0.560	10.250***
Subjective well-being	Gender	0.458	0.450	59.500***	−0.036	−0.033	−1.054
	Marital status				0.025	0.023	0.720
	Income				−0.108	−0.100	−3.057**
	Education level				−0.049	−0.047	−1.447
	Square dance participation				0.125	0.122	2.966**
	Social support				0.174	0.172	3.635**
	Optimism				0.321	0.321	5.944***

***p < 0.001.

In addition, square dance participation significantly predicted social support (β = 0.555, *p* < 0.001), indicating that individuals who participated more frequently in square dance reported higher levels of perceived social support.

Furthermore, both square dance participation (β = 0.034, *p* < 0.001) and social support (β = 0.560, *p* < 0.001) were significant positive predictors of optimism.

When all variables were entered simultaneously into the regression model, square dance participation (β = 0.122, *p* < 0.01), social support (β = 0.172, *p* < 0.01), and optimism (β = 0.321, *p* < 0.001) remained significant predictors of subjective well-being. These results provide preliminary support for the hypothesized mediating roles of social support and optimism.

### 3.4 Mediation analysis

Structural equation modeling (SEM) was conducted using AMOS 26.0 to examine the hypothesized relationships among square dance participation, social support, optimism, and subjective well-being. The results indicated that the model demonstrated a good fit to the data, with χ^2^/df = 1.193, CFI = 0.991, TLI = 0.963, NFI = 0.989, RMSEA = 0.013, and SRMR = 0.044, suggesting that the proposed model was well supported. To examine whether the findings were driven by the highly female-dominated sample, a sensitivity analysis was conducted using the female subsample only. The pattern of associations and mediation effects was consistent with the full-sample results, suggesting that the main findings were not substantially altered when the analysis was restricted to female participants.

As shown in Table 4, square dance participation had a significant positive effect on social support (β = 0.588, C.R. = 8.853, *p* < 0.001), indicating that higher levels of participation were associated with greater perceived social support. Square dance participation also showed a significant positive effect on optimism (β = 0.235, C.R. = 3.367, *p* < 0.001), suggesting that greater square dance participation was associated with higher optimism. In addition, social support was significantly positively associated with optimism (β = 0.407, C.R. = 5.372, *p* < 0.001), supporting Hypothesis 4.

**TABLE 4:** Results of structural equation modeling (SEM).

Path	β	S.E.	C.R.	*p*
Square dance participation → Subjective well-being	0.205	0.069	3.227	0.001
Square dance participation → Social support	0.588	0.051	8.853	< 0.001
Square dance participation → Optimism	0.235	0.064	3.367	< 0.001
Social support → Optimism	0.407	0.089	5.372	< 0.001
Social support → Subjective well-being	0.396	0.104	5.362	< 0.001
Optimism → Subjective well-being	0.145	0.069	2.536	0.011

C.R., critical ratio. All *p* values were calculated using two-tailed tests.

Furthermore, both social support (β = 0.396, C.R. = 5.362, *p* < 0.001) and optimism (β = 0.145, C.R. = 2.536, *p* = 0.011) had significant positive effects on subjective well-being. Finally, square dance participation also had a significant direct effect on subjective well-being (β = 0.205, C.R. = 3.227, *p* = 0.001), supporting Hypothesis 1.

The chain mediating effects of social support and optimism in the relationship between square dance participation and subjective well-being were tested using the bootstrap method in AMOS 26.0, with 5,000 resamples and 95% bias-corrected confidence intervals (CIs). An indirect effect was considered significant if the 95% CI did not include zero.

**FIGURE 2:**
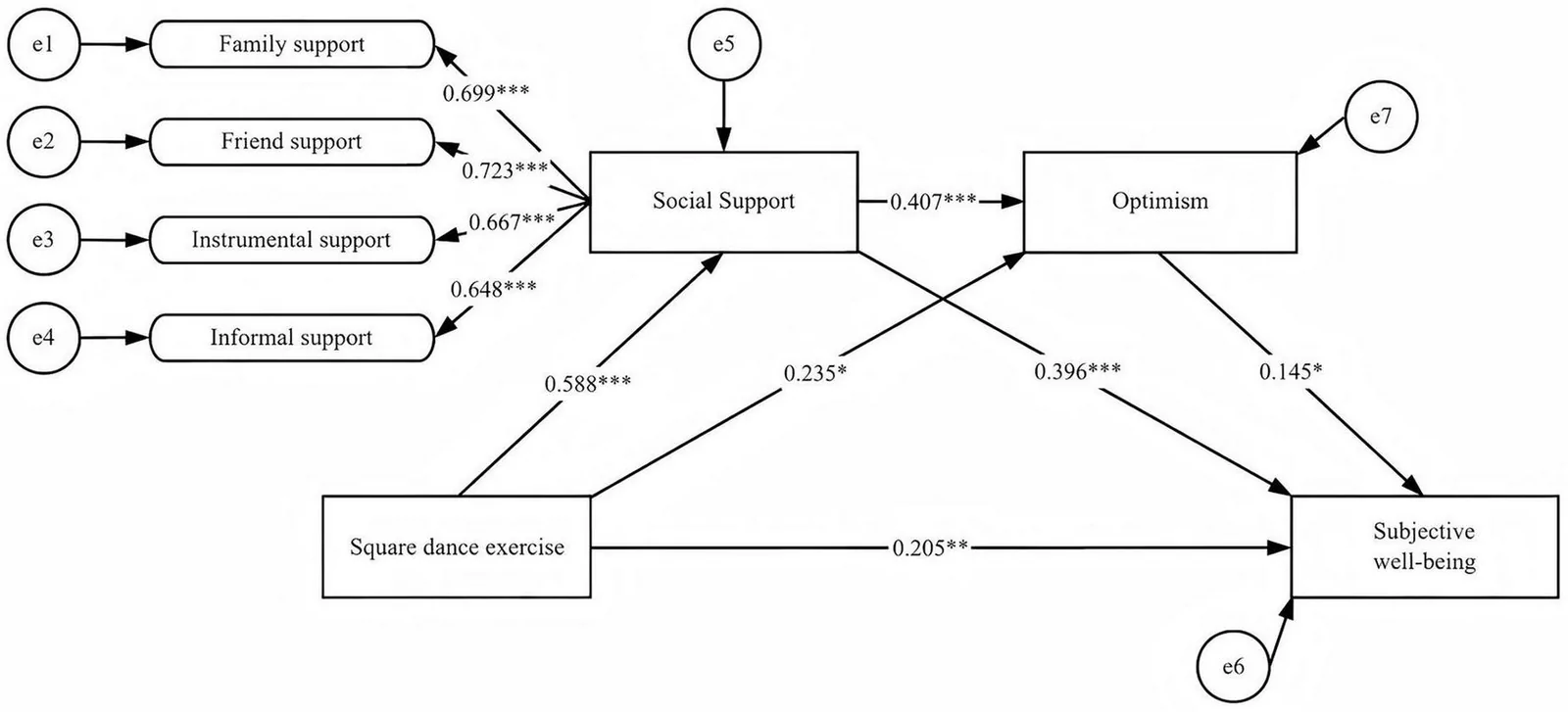
Structural equation model of the relationships among square dance participation, social support, optimism, and subjective well-being. *p < 0.05, **p < 0.01, ***p < 0.001.

As shown in Table 5, square dance participation had a significant indirect effect on subjective well-being through social support (β = 0.233, 95% CI [0.153, 0.336]), accounting for 45.96% of the total effect. The indirect effect through optimism was also significant (β = 0.034, 95% CI [0.005, 0.086]), accounting for 6.71% of the total effect. In addition, the sequential (chain) mediation effect of social support and optimism was significant (β = 0.035, 95% CI [0.008, 0.078]), accounting for 6.90% of the total effect. Collectively, the indirect pathways accounted for 59.57% of the total effect, while the direct effect accounted for 40.43%, indicating a partial mediation pattern. Notably, social support constituted the dominant indirect pathway, explaining over 45% of the total effect, whereas optimism and the sequential pathway together accounted for less than 14%, suggesting a relatively minor contribution of optimism-related mechanisms. The mediation model is illustrated in Figure 2.

**TABLE 5:** Decomposition of total, direct, and indirect effects.

Path	Effect size	SE	95% CI		Proportion (%)
			LLCL	ULCL	
Total effect	0.507	0.043	0.415	0.584	100.00
Direct effect	0.205	0.065	0.077	0.331	40.43
Total indirect effect	0.302	0.042	0.225	0.390	59.57
Indirect 1 (SDP → SS → SWB)	0.233	0.046	0.153	0.336	45.96
Indirect 2 (SDP → OPT → SWB)	0.034	0.020	0.005	0.086	6.71
Indirect 3 (SDP → SS → OPT → SWB)	0.035	0.017	0.008	0.078	6.90

SDP, square dance participation; SS, social support; OPT, optimism; SWB, subjective well-being; CI, confidence interval; LL, lower limit; UL, upper limit. Indirect 1 represents the mediation pathway through social support; Indirect 2 represents the mediation pathway through optimism; Indirect 3 represents the sequential (chain) mediation pathway through social support and optimism.

## 4 Discussion

The present study found that square dance participation was significantly positively associated with subjective well-being among urban older adults, and was also related to higher levels of well-being. This finding is consistent with previous research indicating that engagement in physical activity is positively related to subjective well-being. From the perspective of well-being theory, subjective well-being reflects individuals’ overall evaluation of life satisfaction and emotional experiences, including positive affect and reduced negative affect (Moschis and Mathur, 2024). Physical activity, such as square dance, may be associated with emotional experiences and psychological distress. In line with the definition of health proposed by the World Health Organization, which emphasizes physical, psychological, and social well-being, participation in square dance may be related to physical fitness and mental health. Previous studies have shown that group-based physical activities are associated with reduced negative emotions and better psychological functioning, thereby being related to overall well-being (Chang et al., 2018). Furthermore, square dance is widely practiced in urban communities and is particularly popular among older adults due to its accessibility and social nature (Qu et al., 2023). The present findings highlight square dance as a culturally relevant and practical form of physical activity that may be related to subjective well-being in aging populations. These results provide empirical support for Hypothesis 1 and extend existing literature by showing its association with well-being among urban older adults.

The present study found that social support partially mediated the association between square dance participation and subjective well-being, supporting Hypothesis 2. This suggests that square dance is not only associated with subjective well-being directly but also indirectly through social support. From a need satisfaction perspective, square dance participation may be associated with psychological needs at different levels. At a basic level, physical activity is related to improved physical functioning, which is a foundation of well-being (Steptoe, 1992). At a social level, group-based activities provide opportunities for interaction and may be associated with reduced loneliness and increased interpersonal connection. Square dance is associated with a social context that has been linked to opportunities for social interaction and expanded interpersonal networks among older adults (Butler, 2018). Through participation, individuals may report higher levels of emotional, informational, and instrumental support, which is associated with feelings of belonging and self-worth. This pattern can also be interpreted from conservation of resources theory (Hobfoll, 2009), which conceptualizes social support as an important external resource for psychological well-being. Compared with other psychosocial factors, social support may function as a more stable and immediate resource in later life due to declining personal resources. Therefore, social support may play a relatively more central role in explaining the observed associations between square dance participation and subjective well-being (Zeng and He, 2023). Overall, these findings highlight social support as a key psychosocial pathway statistically linking physical activity and subjective well-being, suggesting that square dance may function not only as physical activity but also as a context associated with social connection and psychological resources.

The present study found that optimism partially mediated the relationship between square dance participation and subjective well-being, supporting Hypothesis 3. This finding is consistent with previous research showing that optimism is associated with well-being. As a positive psychological trait, optimism is related to adaptive coping, positive expectations, and better psychological adjustment (Oishi, 2000; Yarcheski et al., 2004). However, the indirect effect of optimism was relatively small compared with that of social support. One possible explanation is that optimism represents a relatively stable personal disposition, whereas social support may be more directly activated in the context of group-based square dance participation. Square dance provides immediate opportunities for interpersonal interaction and perceived support, while changes in optimism may require longer-term emotional and cognitive processes. Therefore, in this cross-sectional study, optimism may function as a secondary psychological pathway rather than the dominant mechanism linking square dance participation and subjective well-being. Overall, these findings suggest that optimism is a relevant but weaker mediator in this model, highlighting the stronger explanatory role of social support in the association between square dance participation and subjective well-being among urban older adults.

The present study further found a significant sequential mediation effect of social support and optimism, supporting Hypothesis 5. This suggests that the association between square dance participation and subjective well-being may involve both external social resources and internal psychological traits. In this pathway, social support appears to be the dominant component. Square dance, as a group-based activity, provides opportunities for social interaction and community engagement, through which older adults may report higher levels of emotional, informational, and instrumental support. These supportive experiences may be associated with higher levels of optimism (Tripathi, 2017), which is in turn statistically associated with better subjective well-being. However, the relatively small indirect effect of the chain pathway suggests that optimism plays a secondary role compared with social support. This may be because social support is more directly embedded in square dance participation, whereas optimism is a relatively stable psychological trait that may change more gradually. Therefore, the findings highlight social support as the primary psychosocial pathway, with optimism serving as an additional but weaker link in the association between square dance participation and subjective well-being.

## 5 Limitations and prospects

Despite the contributions of this study, several limitations should be acknowledged. First, the sample was limited to a specific urban area, and the proportion of female participants was substantially higher than that of males (94.55%) due to the natural gender composition of square dance participation. This pronounced gender imbalance may limit the generalizability of the findings across different gender groups. Given the small number of male participants, formal gender-stratified comparisons were not appropriate. However, a sensitivity analysis using the female subsample was conducted and showed a pattern broadly consistent with the full-sample results. Future research should recruit more balanced samples or adopt male-oriented, mixed-gender, or intergenerational designs to better examine gender-specific patterns. Second, the cross-sectional design of the study restricts the ability to draw causal inferences. Although the proposed relationships were supported by the data, longitudinal or experimental designs are needed in future research to further examine the causal mechanisms underlying the observed associations. In addition, reverse causality cannot be excluded, whereby individuals with higher levels of subjective well-being, optimism, or social support may be more likely to engage in and maintain square dance participation. Third, due to methodological constraints, the present study examined social support as a composite variable rather than exploring the specific effects of its individual dimensions. Future research could investigate the distinct roles of different types of social support (e.g., family, friend, instrumental, and informational support) in the relationship between physical activity and subjective well-being, as well as their potential differential contributions to the sequential mediation process. Fourth, several potentially important confounding variables were not controlled in the present study, including health status, depression history, living arrangement, and baseline physical activity levels. These factors may influence both square dance participation and subjective well-being. Therefore, the observed mediation effects should be interpreted with caution, as they may be partially influenced by uncontrolled confounding. Furthermore, square dance participation was operationalized using the PARS-3, which primarily measures physical activity volume rather than the social and interactive characteristics of square dance. Future research should incorporate multidimensional measures that better capture both the physical and social aspects of square dance participation. In sum, addressing these limitations in future studies may provide a more comprehensive understanding of the psychosocial mechanisms linking physical activity to well-being among older adults.

## 6 Conclusion

The present study investigated the relationship between square dance participation and subjective well-being among urban older adults, and examined the mediating roles of social support and optimism. The findings revealed that square dance participation was positively associated with subjective well-being, both directly and indirectly through social support and optimism. In particular, a significant sequential (chain) mediation effect was identified, highlighting the joint contribution of social and psychological factors to well-being.

These findings provide theoretical implications for understanding the psychosocial correlates of square dance participation and subjective well-being. At the individual level, square dance participation was associated with subjective well-being, but this association should not be interpreted as evidence of an intervention effect. At the social level, the findings suggest that social support may be an important psychosocial correlate linking group-based physical activity and well-being. Future longitudinal or intervention studies are needed to test whether promoting square dance participation can improve social support, optimism, and subjective well-being among older adults. Group-based physical activities, such as square dance, may serve as effective platforms for fostering social connections and enhancing psychological well-being. At the policy level, community organizations and public health institutions are encouraged to support and promote accessible physical activity programs tailored to older adults. Creating supportive environments that facilitate participation in group-based activities may not only improve physical health but also strengthen social networks and promote positive psychological traits. These efforts are particularly important in the context of population aging and may contribute to improving the overall quality of life among older adults.

## Data Availability

The raw data supporting the conclusions of this article will be made available by the authors, without undue reservation.
